# Oral Manifestations of Wolf-Hirschhorn Syndrome: Genotype-Phenotype Correlation Analysis

**DOI:** 10.3390/jcm9113556

**Published:** 2020-11-04

**Authors:** Jacobo Limeres, Candela Serrano, Joaquin Manuel De Nova, Javier Silvestre-Rangil, Guillermo Machuca, Isabel Maura, Jose Cruz Ruiz-Villandiego, Pedro Diz, Raquel Blanco-Lago, Julian Nevado, Marcio Diniz-Freitas

**Affiliations:** 1 Medical-Surgical Dentistry Research Group (OMEQUI), Health Research Institute of Santiago de Compostela (IDIS), University of Santiago de Compostela (USC), 15782 Santiago de Compostela, Spain; Jacobo.limeres@usc.es (J.L.); cande.s.martin@gmail.com (C.S.); marcio.diniz@usc.es (M.D.-F.); 2 Department of Stomatology IV, School of Dentistry, University Complutense de Madrid, 28040 Madrid, Spain; denova@odon.ucm.es; 3 Department of Stomatology, University Hospital Doctor Peset-FISABIO, 46017 Valencia, Spain; silranja@uv.es; 4 Department of Special Care in Dentistry, School of Dentistry, University of Seville, 41009 Sevilla, Spain; gmachuca@us.es; 5 Service of Pediatric Dentistry, Barcelona University Children’s Hospital HM Nens, 08009 Barcelona, Spain; ismaurasol@hotmail.com; 6 Service of Special Care in Dentistry, Quirón Hospital, 200012 San Sebastián, Spain; ruizvill@mac.com; 7 Service of Neuropediatrics, University Hospital Central de Asturias, 33011 Oviedo, Spain; rablabul@hotmail.com; 8 Medical and Molecular Genetics Institute (INGEMM), La Paz University Hospital, IdiPAZ, 28046 Madrid, Spain; jnevado@salud.madrid.org; 9 Institute of Rare Diseases Research (IIER) & Centre for Biomedical Network Research on Rare Diseases (CIBERER), Instituto de Salud Carlos III, 28029 Madrid, Spain; 10 ERN (European Reference Network)-ITHACA, La Paz University Hospital, 28046 Madrid, Spain

**Keywords:** Wolf-Hirschhorn syndrome, 4p-, stomatognathic diseases, oral manifestations, genotype

## Abstract

Background: Wolf-Hirschhorn syndrome (WHS) is a rare disease caused by deletion in the distal moiety of the short arm of chromosome 4. The objectives of this study were to report the most representative oral findings of WHS, relate them with other clinical characteristics of the disease, and establish possible phenotype-genotype correlation. Methods: The study was conducted at 6 reference centers distributed throughout Spain during 2018–2019. The study group consisted of 31 patients with WHS who underwent a standardized oral examination. Due to behavioral reasons, imaging studies were performed on only 11 of the children 6 years of age or older. All participants had previously undergone a specific medical examination for WHS, during which anatomical, functional, epilepsy-related, and genetic variables were recorded. Results: The most prevalent oral manifestations were delayed tooth eruption (74.1%), bruxism (64.5%), dental agenesis (63.6%), micrognathia (60.0%), oligodontia (45.5%), and downturned corners of the mouth (32.3%). We detected strong correlation between psychomotor delay and oligodontia (*p* = 0.008; Cramér’s V coefficient, 0.75). The size of the deletion was correlated in a statistically significant manner with the presence of oligodontia (*p* = 0.009; point-biserial correlation coefficient, 0.75). Conclusion: Certain oral manifestations prevalent in WHS can form part of the syndrome’s phenotypic variability. A number of the characteristics of WHS, such as psychomotor delay and epilepsy, are correlated with oral findings such as oligodontia and bruxism. Although most genotype-phenotype correlations are currently unknown, most of them seem to be associated with larger deletions, suggesting that some oral-facial candidate genes might be outside the critical WHS region, indicating that WHS is a contiguous gene syndrome.

## 1. Introduction

Wolf-Hirschhorn syndrome (WHS) is a developmental disorder caused by subtelomeric deletion in the distal region of the short arm of chromosome 4 (4p16.3) [1]. Chromosomal microarray techniques showed that a considerable percentage of patients have other cytogenetic aberrations in addition to deletion, such as unbalanced de novo translocations, inverted duplications, and pericentric inversions [2,3,4,5].

**Figure 1:**
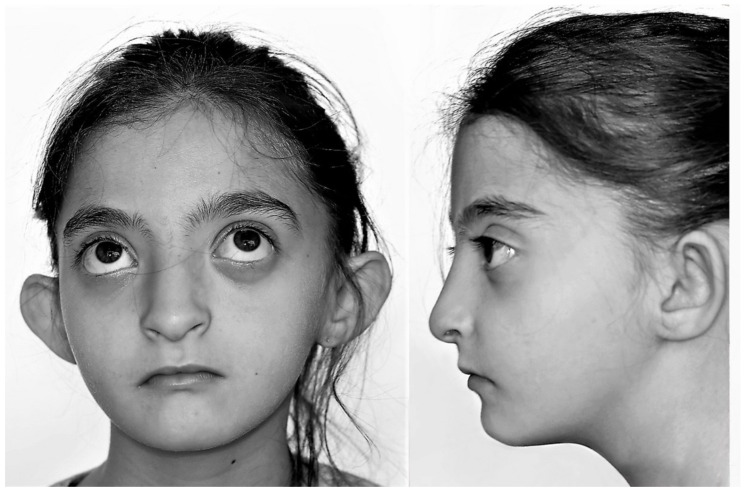
Wolf-Hirschhorn syndrome with characteristic “Greek warrior helmet” facial phenotype.

The disease is considered rare and has an estimated prevalence of 1/50.000 births, with a predominance among the female sex of 2:1 [6]. Although patients with WHS have fragile health for the first years of their life, their mean life expectancy is more than 30 years [7]. To date, more than 300 cases of WHS have been documented in the medical literature, which helped characterize its phenotypic spectrum [7]. An almost pathognomonic finding in these patients is the characteristic “Greek warrior helmet” facies, which consists of a prominent glabella, wide forehead, arched eyebrows, hypertelorism, exophthalmos, epicanthal folds, wide nasal bridge, large nose, short philtrum, micrognathia, and a large mouth with downturned corners (Figure 1) [8]. In addition to this unique facial phenotype, the fundamental manifestations of this polymalformative syndrome in its “classical” presentation include developmental delay, intellectual disability, and epilepsy [9].

The core WHS phenotype is due to the haploinsufficiency of several intimately related genes, which include WHSC1, WHSC2, SLBP, and LETM1 [2,3]. Subsequently, additional genes were identified of which the deletion is necessary for the phenotypic expression to be complete, which include FGFRL1, CPLX1, CTBP1 and PIGG [5,10]. A number of these genes, such as WHSC1 and FGFRL1, are considered candidates for determining the craniofacial phenotype [11,12]. Recent studies based on exome-sequence analysis confirmed that point mutations in the critical NSD2 region (WHSC1) are related to the orofacial phenotype [13] and with numerous other characteristics of the syndrome [14]. However, patients were identified with deletions in the critical WHS region (including WHSC1 and LETM1) who showed none of the characteristic facial features [15]. Correlation between genetic abnormalities and the craniofacial phenotype has, therefore, still not been definitively clarified.

Up to now, the oral manifestations of this syndrome have aroused little interest in the scientific literature. However, and paradoxically, an early and initial suspected diagnosis can be established on the basis of facial traits [16], which requires a thorough examination of the oral cavity. The most numerous series published to date on the general clinical manifestations of WHS (87 patients) indicated that approximately 50% of individuals with this disease have some type of dental abnormality [17]. Nevertheless, most of the available information in the dental literature comes from individual case studies that, in turn, were included in narrative reviews [16,18], except for a series of 7 Finnish patients with WHS in whom the genetic bases of dental agenesis were investigated [19].

The aims of this cross-sectional study were to assess the oral manifestations of WHS in a convenience sample of Spanish patients, and determine whether the oral findings were correlated with other clinical characteristics of WHS, including genotype-phenotype correlations.

## 2. Materials and Methods

### 2.1. Patient Selection

Through the Spanish Wolf-Hirschhorn Syndrome Association (AESWH), we contacted the families of 45 patients with WHS during 2018–2019. The families were informed in writing about the study objectives, and their informed consent was obtained for using the clinical and photographic information. The study was conducted in accordance with the Declaration of Helsinki of the World Medical Association (2008) and approved by the Research Ethics Committee of La Paz University Hospital, Madrid, Spain (PI-2734).

All participants had previously undergone a specific and regulated medical examination for WHS, during which anatomic disorders, clinical findings/comorbidities, developmental abnormalities, and epilepsy-related data were recorded (Table S1). These variables had already been reported and employed for analyzing the Spanish-based cohort of WHS to which the patients in this study belong [4,5,20].

### 2.2. *Single* *Nucleotide Polymorphism (*SNP) Array Analysis

DNA was quantified using PicoGreen (Invitrogen Corporation, Carlsbad, CA, USA). A genomewide scan of 850,000 tag SNPs was conducted at the Medical and Molecular Genetics Institute (INGEMM) using Illumina CytoSNP-850k BeadChip according to the manufacturer’s specifications (Illumina, San Diego, CA, USA). GenCall scores < 0.15 at any locus were considered “no-calls”. Image data were analyzed using the Chromosome Viewer tool contained in Genome Studio (Illumina, San Diego CA, USA). The metric that we employed was the logR ratio, which is the log (base 2) ratio of the observed normalized R value for an SNP divided by the expected normalized R value (under the manufacturer’s specifications). In addition, allele-frequency analysis was applied to all SNPs, and genomic positions were based on GRCh37. We determined the size of the deletion and, in cases in which deletion was associated with duplication, the size of the duplication.

### 2.3. Multiplex Ligation-Dependent Probe Amplification

We employed multiplex ligation-dependent probe amplification (MLPA) kit p096 to determine deletions/duplications in the 4p16.3 region. Reactions were performed according to the manufacturer’s recommendations (MRC Holland, Amsterdam, The Netherlands). Products were analyzed using a fragment analyzer sequencer (ABI 3730XL; Applied Biosystems, Foster City, CA, USA) and 500Liz as the internal size standard. Data analysis was performed using Coffalyser (MRC, Amsterdam, The Netherlans).

### 2.4. Clinical and Radiological Oral Examination

To facilitate their participation, we established a network of 6 reference dental centers distributed strategically throughout Spain (Seville, Madrid, Valencia, Barcelona, San Sebastián, and Santiago de Compostela). A calibration meeting was held with each center’s representatives to record, in a standardized manner, the patients’ dental history, and the results of clinical and radiological oral examination. All patients 6 years and older underwent orthopantomography; cone-beam computed tomography was requested for those patients who presented malformations of the maxillofacial bones or suspected cleft palate.

We collected the following information from each patient: degree of cooperation (based on the number of dental team members necessary to perform the oral examination); examination of the oral cavity with special emphasis on the presence of downturned corners of the mouth, abnormal frenula, cleft lip/palate, ogival palate (high and narrow palate), tooth-eruption chronology, morphological (e.g., peg-shaped teeth, microdontia), numerical (e.g., dental agenesis, oligodontia), and structural (e.g., enamel hypoplasia) tooth abnormalities, and gingival status (basic periodontal examination); intermaxillary relationships and tooth malocclusions in the sagittal, transverse, and vertical planes; and the presence of harmful habits (e.g., bruxism).

### 2.5. Statistical Analysis

We employed the chi-squared test to study the association between 2 nominal categorical variables, and Fisher’s exact test to study the association between 2 dichotomous variables. To determine the magnitude of association between 2 categorical variables, we applied Cramér’s V coefficient (CVC), which is applied to the chi-squared coefficient. We considered that the association between the variables was weak when V was ≤ 0.25, moderate when 0.25 < V < 0.70, and strong when V ≥ 0.70. To quantify the correlation between a dichotomous and a continuous variable, we employed the point-biserial correlation coefficient (CC), a derivative of Pearson’s correlation coefficient. We considered that the association between variables was weak when r < 0.30, moderate when 0.30 ≥ r ≤ 0.70, and strong when r > 0.70. We considered statistically significant only those correlations in which the null hypothesis was rejected in the independence test (*p* value < 0.05) and with a strong degree of association (CVC or CC > 0.70).

## 3. Results

**Figure 2:**
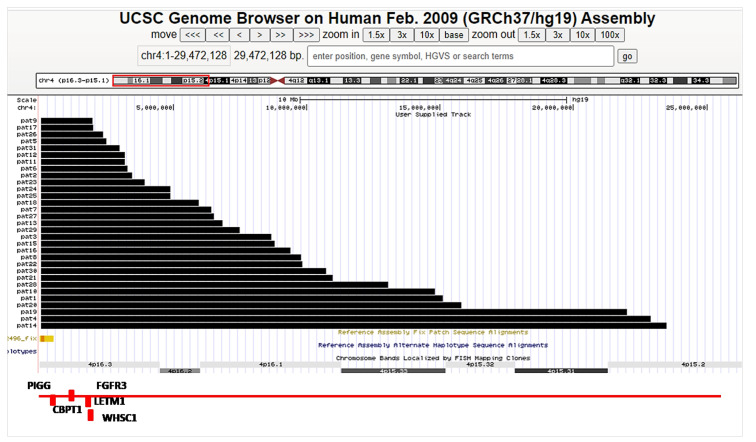
Deletions (black bars) observed in unrelated individuals with Wolf-Hirschhorn syndrome. *, cases with additional rearrangements. Location of 4p cytogenetic bands and known genes of their critical region (#) produced using UCSC genome browser version GRCh37/hg19 (International Human Genome Sequencing Consortium 2004).

The study group consisted of 31 patients (68.8% of all AESWH members), with a mean age of 9.5 ± 3.6 years, with a predominance of the female sex (67.7%). Table 1 lists the participants’ anatomical characteristics and their clinical peculiarities/comorbidities, Table 2 describes the developmental abnormalities and epilepsy characteristics, and Table 3, Figure 2, and Table S2 show the results of the genetic study.

### 3.1. Clinical and Radiological Oral Examination

For 15 patients (48.3%), the oral examination was performed without forceful restraint, 10 (32.2%) required moderate restraint, and 6 (19.3%) required heavy restraint. Of the 23 patients who were 6 years of age or older, imaging studies were performed in only 11 due to behavioral reasons.

Upon examining the oral cavity, we observed that 10 patients (32.3%) had downturned corners of the mouth, and 4 (12.9%) had abnormal frenula. We detected cleft lip/palate in 5 patients (16.1%), and ogival palate in 5 more (16.1%).

**Figure 3:**
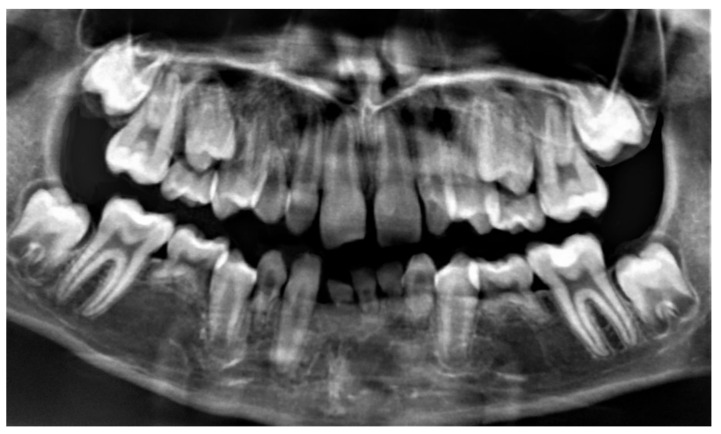
Oligodontia in a patient with Wolf-Hirschhorn syndrome.

With regard to dental abnormalities, 23 patients (74.1%) had delayed tooth eruption, 8 (25.8%) had microdontia, 8 (25.8%) had at least 1 peg-shaped tooth, and 2 (6.4%) had fused teeth. Of the 11 patients whose age and level of cooperation allowed for radiological tests, 7 (63.6%) showed dental agenesis and 5 (45.5%) showed oligodontia (Figure 3). The most affected teeth were the second molars in the temporary teeth and the second premolar in the permanent teeth (Table 4). Radiological findings also included 2 cases (18.1%) of taurodontism. We observed dental attrition in 26 patients (83.8%), caries in 7 (22.5%), and enamel hypoplasia in 3 (9.6%).

Of the 19 patients who were 6 years of age or older and for whom the basic periodontal examination was completed, 4 (21.0%) had healthy gums, 10 (52.6%) had bleeding upon probing, and 5 (26.3%) had bleeding and calculi.

The examination of the intermaxillary relationship could be completed in only 15 patients due to behavioral issues. We diagnosed Angle’s Class II due to micrognathia or retrognathia in 9 (60.0%) patients, crossbite in 5 (33.3%), and overbite in 7 (31.8%) (Table 5). We recorded persistent bruxism in 20 patients (64.5%), 7 (22.5%) had a non-nutritive sucking habit, and 5 (16.1%) presented nibbling.

Oral findings were not correlated with each other except for the downturned corners of the mouth, which was moderately correlated with the presence of a high-arched (ogival) palate (*p* = 0.031; CVC, 0.37).

### 3.2. Correlations between Systemic Findings, Genetic Variables, and Oral Manifestations

We found no statistically significant correlations between anatomical variables and oral findings; however, there was moderate positive correlation between weight and micrognathia/Class II (*p* = 0.040; CC = 0.57), and between height and micrognathia/Class II (*p* = 0.036; CC = 0.58). There was moderate negative correlation between cranial circumference and the presence of abnormal frenula (*p* = 0.003; CC = −0.51; Table S3). We also observed moderate correlation between bruxism and the patient’s sex, which suggests a predominance for the male sex (*p* = 0.042; CVC = 0.37; Table S3).

After analyzing the relationship between clinical findings/comorbidities and oral manifestations, we found no statistically significant correlations; however, we detected a tendency toward correlation between cardiac defects and micrognathia/Angle’s Class II (*p* = 0.035; CVC = 0.69; Table S4).

In terms of developmental abnormalities, we detected strong correlation between psychomotor delay and oligodontia (*p* = 0.008; CVC = 0.75; Table S5).

We found no statistically significant correlations between epilepsy-related data and oral findings, but there was moderate correlation between spasms and abnormal frenula (*p* = 0.013; CVC = 0.68) and between the onset of febrile seizures and crossbite (*p* = 0.035; CVC = 0.69; Table S6). We also found a tendency towards correlation between total number of assayed antiepileptic drugs and bruxism (*p* = 0.065; CVC = 0.45), and a tendency towards positive correlation between degree of seizure control and bruxism (*p* = 0.065; CVC = 0.54).

**Figure 4:**
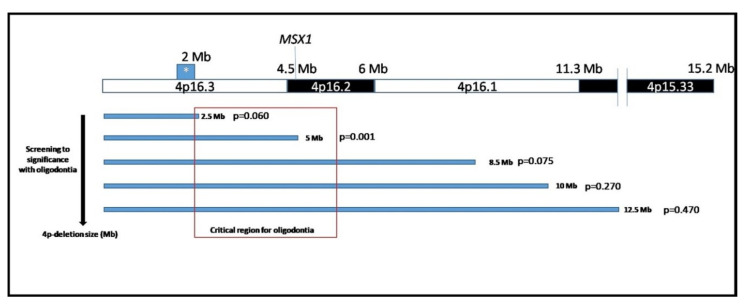
*p*-arm of chromosome-4 screening in Wolf-Hirschhorn syndrome patients with oligodontia. Open square red area is minimal region associated with oligodontia in our cohort (2.3–5.5 Mb). Remarkably, MSX1 gene is placed at 4.85 Mb from telomere.

After analyzing the genetic variables, we found that the size of the deletion was correlated in a statistically significant manner with the presence of oligodontia (*p* = 0.009; CC = 0.75); mean deletion size in individuals with oligodontia was 8.1 ± 3.9 Mb vs. 2.6 ± 0.6 Mb in those without oligodontia. After distributing the deletion sizes into clusters on the basis of clinical manifestations of WHS and a previous classification [3] (< 2.5 >, < 5 >, < 8.5 >, < 10 >, < 12.5 >, and < 13 > Mb), we found statistically significant correlation only when comparing deletions < 5 Mb vs. > 5 Mb in relation to the presence of oligodontia (*p* = 0.001; Figure 4).

There was also moderate correlation between the size of the duplication and crossbite (*p* = 0.047; CC = 0.56), and between size of duplication and bruxism (*p* = 0.014; CC = 0.44; Table S7).

## 4. Discussion

To our knowledge, this is the first study to date of a case series of WHS focused on oral findings and its correlation with the syndrome’s other clinical and genotypic characteristics. The results of anatomical abnormalities, clinical findings/comorbidities, development abnormalities, and epilepsy-related and genetic-study data primarily agree with those obtained in the Spanish-based WHS cohort and were already discussed in previous publications [4,5,20].

In this study, the prevalence of downturned corners of the mouth (1 of every 3 patients) was lower than that reported in previous publications, given that a number of authors it a finding that forms part of the facial-phenotype characteristic of WHS [8,17]. This discrepancy can be affected by various factors such as phenotype severity, observer subjectivity, and the definition of the finding, which in some cases was identified as “distinct mouth” [7,21]. Downturned corners of the mouth are also a frequent finding among patients with chromosome 9q subtelomere deletion syndrome (9qSTDS), one of the most common clinically recognizable syndromes of subtelomere deletions. Individuals with this syndrome also have other characteristics similar to those of patients with WHS, such as gross motor delay, congenital heart defects, and epilepsy [22].

In the literature, studies indicated that abnormal frenula are an uncommon oral manifestation in patients with WHS [18], which has been reported in only an isolated case [6,23]. However, frenulum abnormalities can be an extremely useful indicator in diagnosing a number of syndromic conditions [24]. For example, frenum hyperplasia detected in a number of the patients of the present series was considered an important diagnostic finding in Ellis-van Creveld syndrome (caused by mutations in the EVC1 and EVC2 genes located on chromosome 4p16), which also share other oral manifestations with WHS such as peg-shaped teeth, agenesis, oligodontia, eruption delay, and microdontia [25].

Micrognathia is considered one of the components of the characteristic facial phenotype of WHS [7,17], and our results agree with those in the literature, which estimate a prevalence of > 50% [18]. Micrognathia is a common finding in some of the most common chromosome abnormalities, such as 18p deletion [26]. In micrognathia, the tongue is positioned towards the back and projected in a retrograde direction towards the hard palate, causing a high-arched palate [18]. Cleft lip/palate appears in approximately 25–50% of cases [7], a rate substantially higher than that detected in this series. Considering that isolated cleft palate and high-arched palate might represent the same entity, their joint prevalence would exceed 60% [18]; even assuming this proposal, this rate is surprisingly double that recorded in the present series. Micrognathia, glossoptosis, and respiratory problems constitute the Pierre Robin sequence to which isolated cleft palate was subsequently added. Robin anomalad is related to various genetic syndromes, especially Stickler syndrome [27], followed by Treacher Collins syndrome [28]. MSX1 mutations are observed in the Pierre Robin sequence, and deletion of an MSX1 fragment (4p16.3) located in the WHS critical region can cause a number of the syndrome’s facial characteristics [29].

In 3 of every 4 patients, dental eruption was delayed. The first published reports of WHS suggested that delay in tooth development was a common finding in these patients [30]. Subsequently, the prevalence of delayed dental eruption in WHS was estimated to be 50% [17] and could be clinically identified by the persistence of deciduous teeth [16,17]. Delayed tooth eruption, tooth anomalies, and dental agenesis are also typical findings in patients with 22q11 deletion syndrome who also frequently have congenital heart malformations [31]. We found peg-shaped teeth in 1 of every 4 patients, with the same proportion for microdontia, and almost 1 of every 5 patients who had undergone radiography was diagnosed with taurodontism. Although these abnormalities in tooth size and shape were mentioned in the literature regarding WHS, their prevalence has not been specified [7,17], and most available information corresponds to individual case studies [32,33,34].

In line with our results, other studies suggested that dental agenesis and oligodontia are common findings in WHS [2,19]. Posterior teeth are most often affected [16], especially permanent second premolars [19]. Oligodontia is a finding that also affects approximately half of the patients with Williams syndrome (7q11.23 deletion) [35].

Almost 80% of the patients of the present series had gingival inflammation (bleeding on probing or spontaneous). Although it was suggested that patients with WHS can express some type of immunodeficiency [36,37] that might potentially compromise gingival health, it seems more plausible to attribute this finding to deficient oral hygiene [16], which could also be exacerbated in patients who take hydantoins to control their epilepsy. In a patient group with WHS who had undergone an oral-hygiene regimen under their guardian’s control, there was significant reduction in the rate of gingival inflammation [34].

References regarding malocclusions in WHS are scarce, and a < 10% prevalence was estimated [38]. However, more than half of our patients were Angle’s Class II, presumably due to the micrognathia (mandibular hypoplasia), mandibular retrognathia (abnormal posterior positioning of the mandible), and other predisposing factors such as finger sucking. Mandibular retrognathia is a common finding in patients with 1p36 deletion syndrome—the most common deletion after 22q11.2 deletion [39]—and in patients with 5p deletion syndrome (Cri du chat syndrome) [40]. Malocclusion and crowding are the most frequent dental problems in patients with 22q13 deletion syndrome, also known as Phelan-McDermid syndrome [41].

Bruxism was observed in almost 2 of every 3 patients of the present series, an unprecedented finding, although cases of severely worn dentition in patients with WHS were reported [19]. Its prevalence is predominant in the male sex (9 of 10 male patients vs. 11 of 21 female patients), which suggests that the presence of bruxism could be related to deletion size; however, this hypothesis was confirmed in only the case of duplications. Moreover, the male sex was confirmed to constitute a significant risk factor related to bruxism in the general pediatric population (excluding patients with systemic diseases, syndromes, or neurological or psychiatric disorders [42]. The etiology of bruxism seems to involve certain genes that encode neurotransmitter membrane transport proteins, particularly serotonin and dopamine [43]. In this respect, bruxism and epilepsy in WHS might share a number of genetic abnormalities, as occurs in early infantile epileptic encephalopathy Type 4, which has been related to mutations in the STXBP1 gene, which participates in the pathway of neurotransmitters, including dopamine, and in which 80% of patients have diurnal bruxism [44]. Bruxism in patients with WHS could constitute an indicator of epilepsy severity due to its correlation with the total number of assayed antiepileptic drugs; paradoxically, however, epilepsy control was better among patients with bruxism. The discussion of these findings exceeds the objectives of the present study.

Although the core phenotype of WHS includes atypical facial appearance, mental delay, growth delay, and seizures, phenotypic variability has allowed for patients to be distributed into various categories, which generally correlate with the extent of 4p deletion [3,15]. None of these categories mentions oral manifestations, probably due to the difficulty entailed in diagnosing a number of dental abnormalities, especially in younger patients [19]. Although it appears that frequencies of certain oral manifestations are somewhat higher among patients with single deletions, there were no statistically significant differences with respect to patients with chromosomal rearrangements. This could be due to the limited sample size, but could also be the result of the difference in mean deletion size in the cases of single deletions vs. deletions with duplication (2.5 Mb of difference). There were also no significant differences detected between the two groups in terms of the other variables, such as psychomotor development and epilepsy characteristics. The strict relationship between psychomotor delay (which combines mental delay and growth delay) and oligodontia detected in the present study constitutes new information in the context of WHS. WHSC1 is part of the known critical region for WHS, and is considered the primary gene candidate implicated in global developmental delay [13,14,45]. WHSC1 was also implicated in the development of a number of the syndrome’s facial manifestations [13,45]. Recently, 2 patients who had had hypodontia were reported to have loss-of-function alterations in WHSC1 [14].

Our results indicate that, around the 5 Mb region, there is a gene that is directly involved in oligodontia. MSX1 is a gene that is 4.9 Mb from the telomere [29,46] and represents the primary gene candidate for explaining tooth agenesis [29]. MSX1 haploinsufficiency was shown to be associated with oligodontia in patients with WHS [19], as the results of the present study seem to confirm, although patients who had had oligodontia with terminal deletions smaller than 2.7 Mb were reported [11]. Midline defects that include cleft palate and heart defects were observed in approximately half of WHS cases [46]. The two findings were also related to mutations in the MSX1 gene [47,48], which could help explain the moderate association between heart defects, micrognathia, and oligodontia detected in this study (Table S4). Cleft lip/palate and congenital heart defects appear only in patients with deletions larger than 5 [49] or even 10 Mb [50]. Accordingly, the significant correlation that we encountered between deletion size and oligodontia suggests that genes responsible for tooth agenesis are outside the critical area of WHS.

This study is not exempt from certain limitations. The lack of cooperation from some of the patients hindered the oral examination and/or impeded the radiological study, presumably in those with more severe phenotypes. We found no statistically significant differences in the mean size of 4p-terminal deletions between cooperative patients and those who required moderate/forceful restraint to be able to perform the oral examination (8.5 ± 6.3 and 8.6 ± 6.2 Mb, respectively). However, 26.6% of the cooperative patients had additional rearrangements compared with 68.7% of those who required moderate/forceful restraint. Additionally, the patient group’s cross-sectional character and wide age range could have led to underdiagnosis of certain abnormalities. A number of correlations with a tendency towards statistical significance could have been affected by the restricted sample size.

## 5. Conclusions

Considering the abovementioned limitations of this study, certain oral manifestations prevalent in WHS such as delayed tooth eruption, bruxism, tooth agenesis (especially oligodontia), and micrognathia can form part of the syndrome’s phenotypic variability. In addition, this information can be important for establishing dental requirements of these patients and their management in the dental setting. Correlation between psychomotor delay and oligodontia, and between a number of epilepsy characteristics and oral findings such as bruxism and cross-bite have not been reported to date, and represent new objectives for future investigations. The phenotype-genotype correlation of most of the oral findings is still unknown. Given the limitations of the sample size and the lack of previous publications on this issue, we cannot determine the repercussions of the additional rearrangements on the rate and nature of the syndrome’s oral manifestations. However, the strong association that we found between the size of the 4p deletion and the presence of oligodontia, and between the size of the duplications and bruxism, warrant the search for new candidate genes likely beyond the critical WHS region, which would increase the complexity of the basic genomic defect.

## Figures and Tables

**Table 1:** Anatomical characteristics and clinical findings/comorbidities in study-group patients with Wolf-Hirschhorn syndrome (*n* = 31).

Anatomical Variables			Comorbidities	
Sex	Female	*n* = 21 (67.7%)	Cardiopathy	*n* = 13 (41.9%)
	Male	*n* = 10 (23.2%)	Nephrologic–urologic abnormalities	*n* = 18 (58.1%)
Mean age, years		9.5 ± 3.6 (2.2–20.7)	Ophthalmologic manifestations	*n* = 19 (61.3%)
Weeks of gestation		30.8 ± 3.3 (range 23–39)	Otorhinolaryngologic manifestations	*n* = 15 (48.4%)
Mean weight, g *		1865.0 ± 527.8 (range 800–3440)	Recurrent respiratory infections	*n* = 14 (45.2%)
Mean height, cm *		43.3 ± 3.9 (range 34–52)	Central nervous system malformations	*n* = 15 (48.4%)
Mean cranial circumference, cm *		36.7 ± 2.5 (range 30–41)	Gastrostomy carrier	*n* = 3 (9.7%)
Growth delay		*n* = 22 71.0%	Other surgical history	*n* = 21 (67.7%)

* At birth.

**Table 2:** Developmental abnormalities and epilepsy characteristics of study-group patients with Wolf-Hirschhorn syndrome (*n* = 31).

Developmental Abnormalities		Epilepsy Characteristics	
Head control	*n* = 30 (96.8%)	Diagnosis of epilepsy	*n* = 31 (100%)
Active sitting	*n* = 21 (67.7%)	Age at onset, months	9.9 ± 5.0 (range 0–24)
Walking with support	*n* = 20 (64.5%)	Seizures triggered by fever	*n* = 12 (38.7.0%)
Independent walking	*n* = 11 (35.5%)	Seizures not triggered by fever	*n* = 7 (22.5%)
		Seizures triggered by fever and other conditions	*n* = 12 (38.7%)
Autonomous feeding	*n* = 6 (19.4%)	Status epilepticus	*n* = 14 (45.2%)
			episodes, 3.8 ± 11.0 (range 0–55)
Sphincter control	*n* = 25 (80.6%)	Admission to intensive care due to status epilepticus	*n* = 11 (35.5%)
Interaction with environment	*n* = 28 (90.3%)	Tonic-clonic seizures	*n* = 21 (67.7%)
Communication by gestures/pictograms	*n* = 19 (61.3%)	Atypical absences	*n* = 17 (54.8%)
Emits single words	*n* = 10 (32.3%)	Seizure-free period ≥ 2 years	*n* = 12 (38.7%)
Emits simple phrases	*n* = 4 (12.9%)	AED use	*n* = 26 (83.9%)
Psychomotor development level *	139.1 ± 47.8 (range 36–230)	Uses AEDs in monotherapy	*n* = 17 (54.8%)
Psychomotor delay *	18.4 ± 10.1 (range 1–33)	Valproic acid use	*n* = 16 (51.6%)
Number of comorbidities that affect development	6.8 ± 3.1 (range 1–13)	Levetiracetam use	*n* = 14 (45.1%)

AEDs, antiepileptic drugs; * units listed in Table S1.

**Table 3:** Genetic analysis of study-group patients with Wolf-Hirschhorn syndrome (*n* = 31).

Genetic-Alteration Type		Number of Cases
**Terminal deletions**	Simple 4p- terminal deletions	16 (51.60%)
	4p- terminal deletions and additional terminal duplications in other chromosomes *	13 (41.95%)
	4p- terminal deletions and additional genomic interstitial duplications	2 (6.45%)
**Interstitial deletions**		0 (0%)

* Originated by unbalanced translocations either de novo or inherited. Mean size of 4p- terminal deletions was 8.6 ± 6.1 Mb (range, 1.9–23.5 Mb), and the mean size of additional duplications was 2.3 ± 3.6 Mb (range, 0–15.0).

**Table 4:** Dental agenesis in patients with Wolf-Hirschhorn syndrome.

	Upper Teeth *n* (%)		Lower Teeth *n* (%)	
Temporary teeth *n* = 18	Lateral incisor	2 (11.1)	Lateral incisor	-
	Second molar	8 (44.4)	Second molar	8 (44.4)
Definitive teeth *n* = 47	Central incisor	-	Central incisor	2 (4.2)
	Lateral incisor	8 (17.0)	Lateral incisor	4 (8.5)
	Canine	-	Canine	1 (2.1)
	First premolar	5 (10.6)	First premolar	-
	Second premolar	12 (25.5)	Second premolar	7 (14.9)
	First molar	1 (2.1)	First molar	-
	Second molar	2 (4.2)	Second molar	5 (10.6)

*n*, number of cases of dental agenesis.

**Table 5:** Intermaxillary relationships and tooth malocclusions in patients with Wolf-Hirschhorn syndrome.

Sagittal Plane *n* = 15		Transverse Plane *n* = 15		Vertical Plane *n* = 15	
	*n* (%)		*n* (%)		*n* (%)
Class I	4 (26.6)	Normal occlusion	5 (33.3)	Normal occlusion	6 (40.0)
Class II	9 (60.0)	Crossbite	5 (33.3)	Overbite	7 (46.6)
Class III	2 (13.3)	Scissor bite	3 (20.0)	Open bite	2 (13.3)
Overjet	4 (26.6)				
Anterior crossbite	3 (20.0)				

*n*, number of examined patients; *n*, number of patients who satisfied a specific condition.

## Supplementary Materials

The following are available online at https://www.mdpi.com/2077-0383/9/11/3556/s1. Table S1. Anatomical Variables, Clinical Findings, Developmental Abnormalities, Epilepsy Variables and Genetic Variables Analyzed in the Study Group. Table S2. 4p deletions and rearrangements size in the study group (*n* = 31). Table S3. Association between Anatomical Variables and Oral Findings in Patients with Wolf-Hirschhorn Syndrome. Table S4. Association between Clinical Findings/Comorbidities and Oral Findings in Patients with Wolf-Hirschhorn Syndrome. Table S5. Association between Developmental Abnormalities and Oral Findings in Patients with Wolf-Hirschhorn Syndrome. Table S6. Association between Epilepsy Variables and Oral Findings in Patients with Wolf-Hirschhorn Syndrome. Table S7. Association between Genetic Variables and Oral Findings in Patients with Wolf-Hirschhorn Syndrome.

## References

[1] Battaglia A., South S., Carey J.C. (2011). Clinical utility gene card for: Wolf-Hirschhorn (4p-) syndrome. Eur. J. Hum. Genet..

[2] Maas N.M., Van Vooren S., Hannes F., Van Buggenhout G., Mysliwiec M., Moreau Y., Fagan K., Midro A., Engiz O., Balci S. (2007). The t(4;8) is mediated by homologous recombination between olfactory receptor gene clusters, but other 4p16 translocations occur at random. Genet. Couns..

[3] Zollino M., Murdolo M., Marangi G., Pecile V., Galasso C., Mazzanti L., Neri G. (2008). On the nosology and pathogenesis of Wolf–Hirschhorn Syndrome: Genotype-phenotype correlation analysis of 80 patients and literature review. Am. J. Med. Genet. Part C.

[4] Blanco-Lago R., Malaga-Dieguez I., Granizo-Martinez J.J., Carrera-Garcia L., Barruz-Galian P., Lapunzina P., Nevado-Blanco J. (2017). En Representacion Del Grupo Colaborativo Para El Estudio Del Sindrome de Wolf-Hirschhorn ERDGCPEEDSW. Wolf-Hirschhorn syndrome. Description of a Spanish cohort of 51 cases and a literature review. Rev. Neurol..

[5] Nevado J., Ho K.S., Zollino M., Blanco R., Cobaleda C., Golzio C., Beaudry-Bellefeuille I., Berrocoso S., Limeres J., Barrúz P. (2020). International meeting on Wolf-Hirschhorn syndrome: Update on the nosology and new insights on the pathogenic mechanisms for seizures and growth delay. Am. J. Med. Genet. A.

[6] Lurie I.W., Lazjuk G.I., Ussova Y.I., Presman E.B., Gurevich D.B. (1980). TheWolf- Hirschhorn syndrome. I. Genetics. Clin. Genet..

[7] Battaglia A., Carey J.C., South S.T. (2015). Wolf-Hirschhorn syndrome: A review and update. Am. J. Med. Genet. C. Semin. Med. Gent..

[8] Hammond P., Hannes F., Suttie M., Devriendt K., Vermeesch J.R., Faravelli F., Forzano F., Parekh S., Williams S., McMullan D. (2012). Fine-grained facial phenotype-genotype analysis in Wolf-Hirschhorn syndrome. Eur. J. Hum. Genet..

[9] Zollino M., Di Stefano C., Zampino G., Mastroiacovo P., Wright T.J., Sorge G., Selicorni A., Tenconi R., Zappalà A., Battaglia A. (2000). Genotype-phenotype correlations and clinical diagnostic criteria in Wolf-Hirschhorn syndrome. Am. J. Med. Genet..

[10] Andersen E.F., Carey J.C., Earl D.L., Corzo D., Suttie M., Hammond P., South S.T. (2013). Deletions involving genes WHSC1 and LETM1 may be necessary, but are not sufficient to cause Wolf–Hirschhorn Syndrome. Eur. J. Hum. Genet..

[11] Maas N.M.C., Van Buggenhout G., Hannes F., Thienpont B., Sanlaville D., Kok K., Midro A., Andrieux J., Anderlid B.M., Schoumans J. (2008). Genotype-phenotype correlation in 21 patients with Wolf-Hirschhorn syndrome using high resolution array comparative genome hybridisation (CGH). J. Med. Genet..

[12] Catela C., Bilbao-Cortes D., Slonimsky E., Kratsios P., Rosenthal N., te Welscher P. (2009). Multiple congenital malformations of Wolf–Hirschhorn syndrome are recapitulated in Fgfrl1 null mice. Dis. Model. Mech..

[13] Boczek N.J., Lahner C.A., Nguyen T.M., Ferber M.J., Hasadsri L., Thorland E.C., Niu Z., Gavrilova R.H. (2018). Developmental delay and failure to thrive associated with a loss-of-function variant in WHSC1 (NSD2). Am. J. Med. Genet. A.

[14] Barrie E.S., Alfaro M.P., Pfau R.B., Goff M.J., McBride K.L., Manickam K., Zmuda E.J. (2019). De novo loss-of-function variants in NSD2 ( WHSC1) associate with a subset of Wolf-Hirschhorn syndrome. Cold Spring Harb. Mol. Case Stud..

[15] Yamamoto-Shimojima K., Kouwaki M., Kawashima Y., Itomi K., Momosaki K., Ozasa S., Okamoto N., Yokochi K., Yamamoto T. (2019). Natural histories of patients with Wolf-Hirschhorn syndrome derived from variable chromosomal abnormalities. Congenit. Anom. (Kyoto)..

[16] Paradowska-Stolarz A.M. (2014). Wolf-Hirschhorn Syndrome (WHS)—Literature review on the features of the syndrome. Adv. Clin. Exp. Med..

[17] Battaglia A., Filippi T., Carey J.C. (2008). Update on the clinical features and natural history of Wolf-Hirschhorn (4p-) syndrome: Experience with 87 patients and recommendations for routine health supervision. Am. J. Med. Genet. Part C Semin. Med. Genet..

[18] Morishita M., Shiba R., Chiyo H., Furuyama J., Fujita H., Atsumi Y. (1983). The oral manifestations of 4p- syndrome. J. Oral Maxillofac. Surg..

[19] Nieminen P., Kotilainen J., Aalto Y., Knuutila S., Pirinen S., Thesleff I. (2003). MSX1 gene is deleted in Wolf-Hirschhorn syndrome patients with oligodontia. J. Dent. Res..

[20] Blanco-Lago R., Málaga I., García-Peñas J.J., García-Ron A. (2013). Wolf-Hirschhorn syndrome. A series of 27 patients: Their epidemiological and clinical characteristics. The current situation of the patients and the opinions of their caregivers regarding the diagnostic process. Rev. Neurol..

[21] Chaudhry C., Kaur A., Panigrahi I., Kaur A. (2020). Wolf–Hirschhorn syndrome: A case series from India. Am. J. Med. Genet. Part A..

[22] Stewart D.R., Kleefstra T. (2007). The chromosome 9q subtelomere deletion syndrome. Am. J. Med. Genet. C. Semin. Med. Genet..

[23] Johnson V.P., Mulder R.D., Hosen R. (1976). The Wolf-Hirschhorn (4p-) syndrome. Clin. Genet..

[24] Mintz S.M., Siegel M.A., Seider P.J. (2005). An overview of oral frena and their association with multiple syndromic and nonsyndromic conditions. Oral Surg. Oral Med. Oral Pathol. Oral Radiol. Endod..

[25] Lauritano D., Attuati S., Besana M., Rodilosso G., Quinzi V., Marzo G., Carinci F. (2019). Oral and craniofacial manifestations of Ellis-Van Creveld syndrome: A systematic review. Eur. J. Paediatr. Dent..

[26] Wester U., Bondeson M.L., Edeby C., Annerén G. (2006). Clinical and molecular characterization of individuals with 18p deletion: A genotype-phenotype correlation. Am. J. Med. Genet. A.

[27] Karempelis P., Hagen M., Morrell N., Roby B.B. (2020). Associated syndromes in patients with Pierre Robin Sequence. Int. J. Pediatr. Otorhinolaryngol..

[28] Gomez-Ospina N., Bernstein J.A. (2016). Clinical, cytogenetic, and molecular outcomes in a series of 66 patients with Pierre Robin sequence and literature review: 22q11.2 deletion is less common than other chromosomal anomalies. Am. J. Med. Genet. A.

[29] Paradowska-Stolarz A. (2015). MSX1 gene in the etiology of orofacial deformities. Postepy Hig. Med. Dosw. (Online)..

[30] Miller O.J., Breg W.R., Warburton D., Miller D.A., DeCapoa A., Allderdice P.W., Davis J., Klinger H.P., McGilvray E., Allen F.H. (1970). Partial deletion of the short arm of chromosome no. 4(4p-): Clinical studies in five unrelated patients. J. Pediatr..

[31] Klingberg G., Oskarsdóttir S., Johannesson E.L., Norén J.G. (2002). Oral manifestations in 22q11 deletion syndrome. Int. J. Paediatr. Dent..

[32] Breen G.H. (1998). Taurodontism, an unreported dental finding in Wolf-Hirschhorn (4p-) syndrome. ASDC J. Dent. Child..

[33] Johnston N.J., Franklin D.L. (2006). Dental findings of a child with Wolf-Hirschhorn syndrome. Int. J. Paediatr. Dent..

[34] Dellavia C., Raiteri S., Ottolina P., Pregliasco F. (2011). Oral features in five adult patients with Wolf-Hirschhorn syndrome. Minerva Stomatol..

[35] Castro T., de Paula Martins Saints C., de Oliveira Lira Ortega A., Gallottini M. (2019). Oral characteristics and medical considered in the dental treatment of individual with Williams syndrome. Spec. Care Dentist..

[36] Hanley-Lopez J., Estabrooks L.L., Stiehm R. (1988). Antibody deficiency in Wolf-Hirschhorn syndrome. J. Pediatr..

[37] Campos-Sanchez E., Deleyto-Seldas N., Dominguez V., Carrillo-de-Santa-Pau E., Ura K., Rocha P.P., Kim J.H., Aljoufi A., Esteve-Codina A., Dabad M. (2017). Wolf-Hirschhorn syndrome candidate 1 is necessary for correct hematopoietic and B cell development. Cell. Rep..

[38] Centerwall W.R., Thompson W.P., Allen I.E., Fobes C.D. (1975). Translocation 4p—syndrome: A general review. Am. J. Dis. Child..

[39] Guterman S., Beneteau C., Redon S., Dupont C., Missirian C., Jaeger P., Herve B., Jacquin C., Douet-Guilbert N., Till M. (2019). Prenatal findings in 1p36 deletion syndrome: New cases and a literature review. Prenat. Diagn..

[40] Corcuera-Flores J.R., Casttellanos-Cosano L., Torres-Lagares D., Serrera-Figallo M.A., Rodríguez-Caballero A., Machuca-Portillo G. (2016). A systematic review of the oral and craniofacial manifestations of cri du chat syndrome. Clin. Anat..

[41] Ivanoff C., Ivanoff A. (2014). Deletion syndrome 22q13: What the dentist should know to manage children with Phelan-McDermid syndrome effectively. J. Tenn. Dent. Assoc..

[42] Guo H., Wang T., Niu X., Wang H., Yang W., Qiu J., Yang L. (2018). The risk factor related to bruxism in children: A systematic review and meta-analysis. Arch. Oral. Biol..

[43] Oporto G.H., Bornhardt T., Iturriaga V., Salazar L.A. (2018). Single nucleotide polymorphisms in genes of dopaminergic pathways are associated with bruxism. Clin. Oral. Investig..

[44] Rezazadeh A., Uddin M., Snead O.C., Lira V., Silberberg A., Weiss S., Donner E.J., Zak M., Bradbury L., Scherer S.W. (2019). STXBP1 encephalopathy is associated with awake bruxism. Epilepsy Behav..

[45] Zollino M., Doronzio P.N. (2018). Dissecting the Wolf-Hirschhorn syndrome phenotype: WHSC1 is a neurodevelopmental gene contributing to growth delay, intellectual disability, and to the facial dysmorphism. J. Hum. Genet..

[46] Bergemann A.D., Cole F., Hirschhorn K. (2005). The etiology of Wolf-Hirschhorn syndrome. Trends Genet..

[47] Phan M., Conte F., Khandelwal K.D., Ockeloen C.W., Bartzela T., Kleefstra T., van Bokhoven H., Rubini M., Zhou H., Carels C.E. (2016). Tooth agenesis and orofacial clefting: Genetic brothers in arms?. Hum. Genet..

[48] Li F.F., Han Y., Shi S., Li X., Zhu X.D., Zhou J., Shao Q.L., Li X.Q., Liu S.L. (2015). Characterization of transcriptional repressor gene MSX1 variations for possible associations with congenital heart diseases. PLoS ONE.

[49] Yang W.X., Pan H., Li L., Wu H.R., Wang S.T., Bao X.H., Jiang Y.W., Qi Y. (2016). Analyses of genotypes and phenotypes of ten Chinese patients with Wolf-Hirschhorn syndrome by multiplex ligation-dependent probe amplification and array comparative genomic hybridization. Chin. Med. J. (Engl.).

[50] Wieczorek D., Krause M., Majewski F., Albrecht B., Horn D., Riess O., Gillessen-Kaesbach G. (2000). Effect of the size of the deletion and clinical manifestation in Wolf-Hirschhorn syndrome: Analysis of 13 patients with a de novo deletion. Eur. J. Hum. Genet..

