# Supplementary material for: Oral Manifestations of Wolf-Hirschhorn Syndrome: Genotype-Phenotype Correlation Analysis
Source: J Clin Med. 2020 Nov 4;9(11):3556. doi: 10.3390/jcm9113556 (PMC7694380; doi:10.3390/jcm9113556)
Supplement: Supplementary file 1 [file jcm-09-03556-s001.pdf]

Journal of
Clinical Medicine

MDPI

Table S1. Anatomical Variables, Clinical Findings, Developmental Abnormalities, Epilepsy Variables and Genetic Variables Analyzed in the Study Group.

Type of Variable	Description of the Variable	Assessment
	Sex	Female/Male
	Weeks of gestation	Weeks
**Anatomical**	Birth weight	Grams
	Birth height	Cm
	Cranial circumference at birth	Cm
	Growth delay	Yes/No
	Cardiopathy	Yes/No
	Nephrologic-urologic abnormalities	Yes/No
	Ophthalmologic manifestations	Yes/No
**Clinical findings/comorbidities**	Otorhinolaryngologic manifestations	Yes/No
	Recurrent respiratory infections	Yes/No
	Central nervous system malformations	Yes/No
	Gastrostomy carrier	Yes/No
	Other surgical history	Arbitrary scale (0–4)
	Head control	Yes/No
	Active sitting	Yes/No
	Walking with support	Yes/No
	Independent walking	Yes/No
	Autonomous for feeding	Yes/No
	Sphincter control	Yes/No
	Interaction with the environment	Yes/No
**Developmental abnormalities**	Communication by gestures/pictograms	Yes/No
	Emits single words	Yes/No
	Emits simple phrases	Yes/No
	Motor delay	Arbitrary scale (0–6)
	Cognitive delay	Arbitrary scale (0–5)
	Psychomotor development level	Haizea-Llevant numerical scale (97 items)*
	Psychomotor delay	Age-adjusted development level
	Comorbidities that affect development	Numerical scale
	Diagnosis of epilepsy	Yes/No
	Age at onset	Months
	Seizures triggered by fever	Yes/No
	Seizures not triggered by fever	Yes/No
	Status epilepticus	Yes/No
	Admission to intensive care due to status epilepticus	Yes/No
	Generalized tonic-clonic seizures	Yes/No
	Myoclonic seizures	Yes/No
	Partial seizures	Yes/No
**Epilepsy variables**	Atypical absence seizures	Yes/No
	Spasms	Yes/No
	Tonic seizures	Yes/No
	AED use	Yes/No
	Uses AEDs in monotherapy	Yes/No
	Total number of AEDs that have been assayed	Arbitrary scale (0–5)
	Maximum number of AEDs consumed simultaneously	Arbitrary scale (0–3)
	Seizure control	Arbitrary scale in months since the last seizure (0–6)
	Overall patient assessment	Numerical scale**
**Genetic variables**	Deletion size	Megabases

	Deletion size	Megabases

*Anonymous. Haizea-Llevant Study. Vitoria: Central Publications Department. Basque Government eds.; 1991; AEDs,antiepileptic drugs; ** Sum of 4 epilepsy variables, comorbidities, developmental level, and psychomotor delay.

Table S2. 4p deletions and rearrangements size in the study group (n = 31).

Patient Number	4p Deletion Size (Mb)	Extra Rearrangement Chr (Band)/Type	Extra Rearrangement Size (Mb)	Extra Second Rearrangement Chr/Type	Extra Second Rearrangement size (Mb)
1	15.1	-	-	-	-
2	3.45	-	-	-	-
3	8.67	8p23.3-23.1/duplication	6.7	-	-
4	22.9	-	-	-	-
5	2.5	-	-	-	-
6	3.3	8p23.3-23.1/duplication	6.6	-	-
7	6,2	-	-	-	-
8	9.8	-	-	-	-
9	1.99	11p15.5-15.4/duplication	3.5	-	-
10	14,8	-	-	-	-
11	3.2	20p13/duplication	1.35	-	-
12	3.19	20p13/duplication	1.37	-	-
13	6.84	-	-	-	-
14	23.5	8p23.3-23.1/duplication	7.37	-	-
15	8,8	8p23.3-23.1/duplication	6.5	-	-
16	9.4	8p23.3-23.1/duplication	7	-	-
17	2.01	-	-	-	-
18	5.95	-	-	-	-
19	22	-	-	-	-
20	15.8	1q42.3-q44/duplication	15.04	1	-
21	10.98	-	-	-	-
22	9.85	-	-	-	-
23	3.94	12p13.31-13.33/duplication	8.39	-	-
24	4.9	22q13.3/duplication	2.1	-	-
25	4.9	11p15.5/duplication	0.5	-	-
26	2.38	14q11.2/duplication (int)	1	14q32.33/duplication (int)	0.187
27	6.52	5q21.1/duplication (int)	0.85	-	-
28	13.06	-	-	-	-
29	7.49	-	-	-	-
30	10.73	10q26.3/duplication	3.55	-	-

31	2.99	-	-	-	-

Table S3. Association between Anatomical Variables and Oral Findings in Patients with Wolf-Hirschhorn Syndrome.

	**Downward Oral Commissures**	**Abnormal Frenula**	**Cleft Palate**	**Ogival Palate**	**Cleft or Ogival Palate**	**Delayed Eruption**	**Peg-Shaped Teeth**	**Microdontia**	**Dental Agenesis**	**Oligodontia**	**Micrognathia / Class II**	**Crossbite**	**Bruxism**
Sex	0.11	0.15	0.06	0.00	0.06 (0.007)	0.22	0.09	0.07	0.13	0.04	0.43	0.10	0.37
Weeks of gestation	-0.09	-0.23	-0.41	-0.21	0.13	0.29	-0.19	0.13	-0.14	-0.25	0.41	-0.18	-0.05
Weight*	0.02	-0.23	-0.43 (0.018)	-0.19	0.14	0.28	-0.29	0.21	0.13	-0.03	0.57 (0.040)	-0.28	-0.07
Height*	0.17	-0.36 (0.045)	-0.19	-0.08	0.03	0.37 (0.038)	-0.22	0.14	0.06	0.02	0.58 (0.036)	-0.32	0.06
Cranial circumference*	0.07	-0.51 (0.003)	-0.08	-0.02	0.02	0.48 (0.007)	-0.14	-0.08	-0.07	-0.33	0.33	-0.43	-0.10
Growth delay	0.17	0.03 (0.035)	0.29	0.11	0.10	0.21	0.11	0.43	0.21	0.07	0.03	0.41	0.12

The white cells contain the values of Cramer's V coefficient; the grey cells contain the values of the point-biserial correlation coefficient; the parentheses contain the p-values <0.05; * At birth.

Table S4. Association between Clinical Findings/Comorbidities and Oral Findings in Patients with Wolf-Hirschhorn Syndrome.

	Downward Oral Commissures	Abnormal Frenula	Cleft Palate	Ogival Palate	Cleft or Ogival Palate	Delayed Eruption	Peg-Shaped Teeth	Microdontia	Dental Agenesis	Oligodontia	Micrognathia / Class II	Crossbite	Bruxism
Cardiopathy	0.03 (0.034)	0.26	0.18	0.09	0.18	0.25	0.10	0.10	0.46	0.67	0.69 (0.035)	0.32	0.08
Nephrologic-urologic abnormalities	0.03	0.13	0.03	0.16	0.15	0.10	0.10	0.10	0.07	0.10	0.10	0.16	0.33
Ophthalmologic manifestations	0.27	0.09	0.37	0.24	0.00	0.17	0.01	0.14	0.45	0.47	0.03	0.03	0.04
Otorhinolaryngologic manifestations	0.12	0.20	0.12	0.12	0.12	0.31	0.31	0.17	0.04	0.26	0.35	0.22	0.31
Recurrent respiratory infections	0.07	0.04	0.33	0.01	0.21	0.06	0.06	0.06	0.07	0.10	0.22	0.30	0.00
Central nervous system malformations	0.02	0.20	0.27	0.07	0.09	0.17	0.31	0.31	0.24	0.35	N/A	N/A	0.18
Gastrostomy carrier	0.24	0.13	0.45	0.02	0.15	0.06	0.06	0.06	0.18	0.45	0.50	0.35	0.01
Other surgical history	0.37	0.37	0.50	0.36	0.25	0.21	0.21	0.31	N/A	N/A	N/A	N/A	0.48

The cells contain Cramér's V coefficient values; the parentheses contain the p-values <0.05; N/A, not applicable.

Table S5. Association between Developmental Abnormalities and Oral Findings in Patients with Wolf-Hirschhorn Syndrome.

	Downward Oral Commissures	Abnormal Frenula	Cleft Palate	Ogival Palate	Cleft or Ogival Palate	Delayed Eruption	Peg-Shaped Teeth	Microdontia	Dental Agenesis	Oligodontia	Micrognathia / Class II	Crossbite	Bruxism
Head control	0.13	0.07	0.08	0.12	0.08	0.11	0.31	0.31	N/A	N/A	N/A	N/A	0.25
Active sitting	0.03	0.35	0.25	0.00	0.13	0.07	0.54	0.09	0.24	0.35	0.10	0.10	0.22
Walking with support	0.07	0.32	0.22	0.05	0.15	0.13	0.49	0.02	0.24	0.35	0.10	0.10	0.13
Independent walking	0.07	0.29	0.32	0.15	0.06	0.02	0.28	0.18	0.36	0.52	0.03	0.30	0.01
Autonomous for feeding	0.19	0.19	0.20	0.10	0.04	0.29	0.29	0.08	0.31	0.10	0.18	0.43	0.02
Sphincter control	0.19	0.19	0.20	0.10	0.04	0.29	0.29	0.08	0.07	0.10	0.16	0.43	0.19
Interaction with the environment	0.01	0.20	0.15	0.02	0.15	0.19	0.06	0.06	N/A	N/A	0.23	0.23	0.01
Communication with gestures and pictograms	0.27	0.09	0.00	0.06	0.18	0.14	0.29	0.01	0.36	0.52	0.16	0.22	0.04
Emits single words	0.03	0.27	0.32	0.31	0.13	0.09	0.41	0.07	0.31	0.63	0.10	0.53	0.07
Emits simple phrases	0.06	0.15	0.18	0.04	0.09	0.01	0.23	0.23	0.04	0.56	0.06	0.34	0.32
Motor delay	0.31	0.43	0.46	0.41	0.23	0.54	0.62	0.57	N/A	N/A	N/A	N/A	0.32
Cognitive delay	0.39	0.37	0.26	0.37	0.50	0.27	0.37	0.38	N/A	N/A	0.59	N/A	0.28
Psychomotor development level	0.03	0.26	0.35	0.12	-0.03	-0.36	0.12	-0.04	0.06	0.31	-0.48	0.47	0.24
Psychomotor delay	0.01	0.36 (0.018)	0.48	0.32	0.02	-0.09 (0.014)	0.46 (0.012)	-0.09	0.38	0.75 (0.008)	-0.28	0.36	0.18
Comorbidities that affect development	0.09	0.12	0.43	0.12	-0.10	-0.20	0.08	-0.06	0.37	0.23	-0.23	0.31	0.23

The white cells contain the values of Cramér’s V coefficient; the grey cells contain the values of the point-biserial correlation coefficient; the parentheses contain the p-values <0.05; N/A, not applicable.

Table S6. Association between Epilepsy Variables and Oral Findings in Patients with Wolf-Hirschhorn Syndrome.

	Downward Oral Commissures	Abnormal Frenula	Cleft Palate	Ogival Palate	Cleft or Ogival Palate	Delayed Eruption	Peg-Shaped Teeth	Microdontia	Dental Agenesis	Oligodontia	Micrognathia / Class II	Crossbite	Bruxism
Diagnosis of epilepsy	0.13	0.07	0.08	0.12	0.08	0.11	0.31	0.31	N/A	N/A	N/A	N/A	0.25
Age at onset	0.11	-0.3	-0.22	-0.12	0.06	0.04	-0.39	0.09	-0.31	-0.17	-0.05	-0.34	0.08
Seizures triggered by fever	0.14	0.39	0.10	0.05	0.10	0.05	0.27	0.11	0.24	0.35	0.34	0.69 (0.035)	0.18
Seizures not triggered by fever	0.11	0.13	0.03	0.13	0.21	0.25	0.05	0.10	0.21	0.31	0.30	0.18	0.05
Status epilepticus	0.07	0.04	0.15	0.16	0.15	0.09	0.21	0.06	0.46	0.26	0.16	0.22	0.13
Intensive Care	0.07	0.12	0.03	0.11	0.22	0.02	0.18	0.18	0.46	0.26	0.16	0.10	0.13
Generalized tonic-clonic seizures	0.03	0.15	0.13	0.15	0.13	0.09	0.07	0.22	0.18	0.07	0.53	0.54	0.07
Myoclonic seizures	0.21	0.09	0.17	0.19	0.10	0.14	0.12	0.29 (0.032)	0.04	0.26	0.03	0.10	0.19
Partial seizures	0.11	0.06	0.25	0.31	0.06	0.09	0.09	0.41 (0.032)	0.04	0.26	0.03	0.10	0.21
Atypical absence seizures	0.07	0.16	0.24	0.17	0.06	0.24	0.24	0.09	0.45	0.47	0.10	0.30	0.14
Spasms	0.18	0.68 (0.013)	0.12	0.17	0.12	0.15	0.15	0.15	0.24	0.35	0.37	0.37	0.19
Tonic seizures	0.29	0.18	0.27	0.39	0.27	0.05	0.05	0.11	0.07	0.10	0.16	0.06	0.12
AED use	0.11	0.09	0.20	0.10	0.04	0.34	0.14	0.34	0.39	0.15	0.16	0.32	0.04
Uses AEDs in monotherapy	0.21	0.42 (0.032)	0.15	0.13	0.21	0.21	0.06	0.09	0.46	0.26	0.10	0.03	0.00
Total number of AEDs that have been assayed	0.33	0.48	0.43	0.38	0.48	0.37	0.41	0.20	N/A	N/A	N/A	N/A	0.45
Maximum number of AEDs	0.28	0.41	0.15	0.40	0.38	0.34	0.22	0.17	N/A	N/A	0.25	0.53	0.11
Seizure control	0.32	0.61	0.54	0.48	0.54	0.53	0.40	0.56	N/A	N/A	N/A	N/A	0.54
Overall patient assessment	0.02	0.27	0.40	0.21	0.03	-0.19	0.20	-0.10	0.23	0.45	-0.40	0.27	0.22

The white cells contain the values of Cramer's V coefficient; the grey cells contain the values of the point-biserial correlation coefficient; the parentheses contain the p-values <0.05; N/A, not applicable; Intensive care, Admission to intensive care due to status epilepticus; AEDs, antiepileptic drugs; maximum number of AEDs, maximum number of AEDs consumed simultaneously.

Table S7. Association between Genetic Variables and Oral Findings in Patients with Wolf-Hirschhorn Syndrome.

	Downward Oral Commissures	Abnormal Frenula	Cleft Palate	Ogival Palate	Cleft or Ogival Palate	Delayed Eruption	Peg-Shaped Teeth	Microdontia	Dental Agenesis	Oligodontia	Micrognathia / Class II	Crossbite	Bruxism
Deletion size	0.20	0.05	0.36	0.26	0.06	-0.04	0.28	-0.18	0.51	0.74 (0.009)	0.12	0.22	0.21
Deletion size	-0.31	0.01	0.12	-0.25	-0.09	-0.34	0.25	0.09	-0.28	-0.06	-0.21	0.56 (0.047)	0.44 (0.014)

The grey cells contain the values of the point-biserial correlation coefficient; the parentheses contain the p-values <0.05.
